# Leopard Density Estimation within an Enclosed Reserve, Namibia Using Spatially Explicit Capture-Recapture Models

**DOI:** 10.3390/ani9100724

**Published:** 2019-09-25

**Authors:** Jenny Noack, Louis Heyns, Diethardt Rodenwoldt, Sarah Edwards

**Affiliations:** AfriCat Foundation, Otjiwarongo P.O. Box 1889, Namibia; louis@africat.org (L.H.); africatvet@africat.org (D.R.); sarah@africat.org (S.E.)

**Keywords:** conservation, density, enclosed reserve, leopard, *Panthera pardus*, spatially explicit capture-recapture

## Abstract

**Simple Summary:**

Due to continuous levels of human–wildlife conflict, habitat loss and fragmentation, the establishment of protected and enclosed reserves constitute a solid foundation for the long-term survival of threatened species. Because species living in enclosed systems often behave differently compared to their free-roaming counterparts, research is forming an important and essential tool to understand their ecology and behavior. For a population to be sustainable in a closed, fenced system, effective conservation and management strategies need to be developed on the basis of robust population estimates. We found that the study area, a protected nature reserve, is harbouring the highest leopard density in Namibia to date, highlighting that small, enclosed reserves can play a vital role for the survival of threatened species in the future.

**Abstract:**

The establishment of enclosed conservation areas are claimed to be the driving force for the long-term survival of wildlife populations. Whilst fencing provides an important tool in conservation, it simultaneously represents a controversial matter as it stops natural migration processes, which could ultimately lead to inbreeding, a decline in genetic diversity and local extinction if not managed correctly. Thus, wildlife residing in enclosed reserves requires effective conservation and management strategies, which are strongly reliant on robust population estimates. Here, we used camera traps combined with the relatively new class of spatially explicit capture-recaptured models (SECR) to produce the first reliable leopard population estimate for an enclosed reserve in Namibia. Leopard density was estimated at 14.51 leopards/100 km^2^, the highest recorded density in Namibia to date. A combination of high prey abundance, the absence of human persecution and a lack of top-down control are believed to be the main drivers of the recorded high leopard population. Our results add to the growing body of literature which suggests enclosed reserves have the potential to harbour high densities and highlight the importance of such reserves for the survival of threatened species in the future.

## 1. Introduction

The establishment of protected areas like national parks and private game reserves is claimed to be the driving force for the long-term survival of wildlife populations and the preservation of biodiversity [1]. Protected areas in southern Africa are often surrounded by electrical boundary fences which separate protected areas from areas influenced by anthropogenic activity and thus, mitigate the risk of edge effects, especially poaching [2]. While fencing provides an important tool in conservation, it represents a controversial matter as impermeable fencing stops natural processes such as emigration and immigration for some species [3] and thus negatively impacts genetic viability. Small fenced reserves often require intensive population management for large species. Robust estimations of carnivore populations are critically important and have a valuable input in forming effective conservation and management strategies for fenced reserves.

Leopards (*Panthera pardus*) are classified as ‘Vulnerable’ by the IUCN (International Union for Conservation of Nature) with a decreasing population trend [4]. Leopards occurred historically throughout Namibia; however, they are currently absent from about 30% of their historic range [5,6]. The majority of leopards occurring outside of protected areas are increasingly involved with real or perceived conflict with livestock and game farmers. As human–wildlife conflict and habitat loss and fragmentation are the main drivers of the population decline in Namibia, leopards could benefit from the presence of protected reserves with reduced anthropogenic influence. 

Here, we estimate the density of a naturally occurring leopard population using camera traps combined with the relatively new class of spatially explicit capture-recaptured models (SECR), in a protected and enclosed reserve in central Namibia. The application of camera traps within the study area enhances previous research efforts in the study area, namely of very high frequency (VHF) radio collars on a select number of individuals, as camera traps allow for the monitoring of the entire leopard population. Due to the presence of the fence which is securing a stable, year-round food resource and the absence of human persecution, we hypothesize that the density of leopards will be higher in the study area compared to densities previously estimated for open systems in Namibia. 

## 2. Materials and Methods 

### 2.1. Study Area

The study was conducted in the Okonjima Nature Reserve (ONR) in central Namibia. The privately-owned reserve compromises a total size of 200 km^2^ and is entirely enclosed by a 2.4-m-high electrified fence bordering commercial farmland on all sides. The reserve is used for eco-tourism and high-end photographic safaris. No hunting activities are carried out in the reserve. Tourism lodges as well as the headquarters of the AfriCat Foundation and staff housing are situated in the southern part of the reserve, which is additionally fenced off creating a 20 km^2^ area which is considered to be leopard-free. Leopards occur naturally in the reserve and are considered the apex predator. Other carnivores present in the ONR during the study period included cheetah (*Acinonyx jubatus*), wild dog (*Lycaon pictus*), brown hyaena (*Parahyaena brunnea*) and spotted hyaena (*Crocuta crocuta*). Lions (*Panthera leo*) are absent from the study area. Herbivore densities are unnaturally high in the study area, as recorded during an aerial game survey in 2018, with for example 244 kudu (*Tragelaphus strepsiceros*)/100 km^2^, 290 impala (*Aepyceros melampus*)/100 km^2^ and 420 gemsbok (*Orxy gazella*/100 km^2^. In contrast, game numbers on farmland surrounding the Waterberg Plateau Park in north-central Namibia were estimated at 8.8/100 km^2^ [7]. Previous research in ONR has estimated a density of 24.0 brown hyaena/100 km^2^, the highest recorded density for the species from anywhere within its distribution [8].

### 2.2. Data Collection

**Figure 1:**
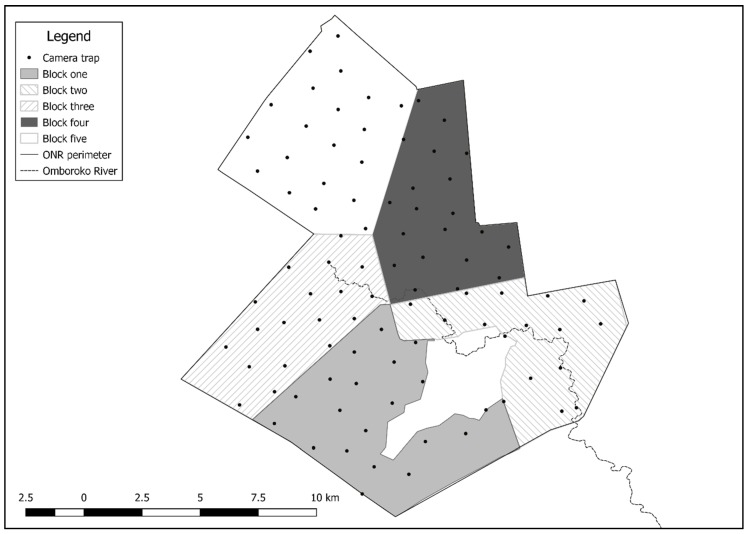
Division of the study area in five blocks and camera trap placement used for leopard density estimation in Okonjima Nature Reserve (ONR).

Due to a limited number of camera traps available during the survey period, leopard density was estimated using a ‘blocked’ survey design [9], in which the part of the ONR in which leopards occurred was divided into five blocks, approximately 40 km^2^ each (Figure 1). The number of camera trap stations within each block varied from 16 to 20 (block one *n* = 20, block two *n* = 16, block three *n* = 19, block four *n* = 20, block five *n* = 20), and stations were active in each block for a total of 50 trap nights (24 July 2015–11 May 2016). Camera trap stations were placed in areas known to be frequented by leopards (dry river beds, riverbanks and game trails), at a mean nearest neighbour distance of 1.19 km (SD = 0.23), and consisted of a single Cuddeback X-change white flash camera trap model 1279 (Non Typical Inc., Park Falls, WI, USA), housed in a Cuddesafe protective housing. Camera stations were baited as the method has shown to increase both leopard capture frequency and probability, whilst not influencing spatial movements [10]. Bait consisted of a quarter or half donkey head secured to a tree with wire, and were placed ~1.75 metres above ground to try to ensure that only leopards interacted with the bait. Camera traps were aimed at the bait and programmed to take three photos per trigger, with no delay between triggers at a photo quality of 5 mega pixel (MP); traps were visited once a week to renew bait and change SD cards and batteries. 

Each individually identified leopard was assigned with an identification number and age and sex class determined. Due to the ongoing long-term monitoring of leopards in the ONR, the age of the majority of individuals was known prior to the study. Unknown individuals were classified based on their size. 

### 2.3. Density Estimations

To estimate density, the ‘secr’ package [11] in statistical program R [12] was used. The package estimates density using a maximum likelihood spatially explicit capture-recapture (SECR) framework, by combining individual capture histories with the location each individual was detected [13]. This relatively new class of models overcomes the problem of defining the sampling area associated with traditional non-spatial models, in which the resulting abundance estimate is converted into density ad hoc [14]. Along with a density estimate, ‘secr’ produces an estimate of g0 (λ_0_), the probability of detection at the centre of a home range, as well as of sigma (σ), a function of the scale of animal movement. Models can be ran in which λ_0_ and σ are influenced by various factors.

Within package ‘secr’, models were ran using the hazard rate detection (observational) process, as this is most appropriate for situations in which the study area is surrounded by a natural or artificial boundary, given that density estimates from such situations do not reach a plateau fairly promptly with an increasing buffer width [13]. As the exact trapping area was known, and during the survey no leopard were recorded passing through the fence (as monitored using VHF collars (*n* = 13) and camera traps (*n* = 10) that were placed outside of the ONR boundary fence), the fenced area was used as the state space. Only adult and independent sub-adults (i.e., sub-adult individuals which visited bait stations alone) were included with density estimation models. Six models were ran: (1) a null model (λ_0_~1, σ~1) in which both g0 and sigma were constant; (2) a behaviour model (λ_0_~b, σ~1) in which g0 was affected by a reaction of individuals to camera traps, i.e., the detection of probability changes as a result of the initial encounter, often referred to as ‘trap happy’ or ‘trap shy’ (λ_0_~b, σ~1). (3) A second behaviour model (λ_0_~b, σ~b) named as the behavioural response *b_2_*_,_ in which reaction of individuals to camera traps affects both g0 and sigma; and (4) a sex λ_0_ model (λ_0_~sex, σ~1) in which λ_0_ was influenced by leopard sex; (5) a sex σ model (λ_0_~1, σ~sex) in which σ was influenced by leopard sex and (6) a full sex model (λ_0_~sex, σ~sex) in which both λ_0_ and σ are influenced by sex. Model fit was ranked using Akaike information criterion (AIC) values [15], to indicate the level of support for each model [16]. Population closure was assessed using the closure test within the ‘secr’ package [17]. 

## 3. Results

### 3.1. General Descriptives

The total sampling effort accumulated 4566 trap nights and resulted in a total number of 36 captured leopards including 24 adults (12 males, 12 females), five sub-adults (three males, two females) and seven juvenile (≤18 months) individuals. All sub-adult individuals were recorded visiting bait stations alone and thus were classed as independent and included within density estimates. Leopards were captured a total of 457 times expanded over 250 sampling occasions defined as a 24-h period of active camera trap days. Capture frequencies ranged from one–twelve captures per individual leopard (12.1 ± 8.1). Leopards were photographed at 90 out of the 95 camera stations. The population closure test indicated no violation of the population closure assumption (*z* = −2.78, *p* = 0.04).

### 3.2. Density Estimation

The full sex model was deemed to be the best fitting model. As all other models had ∆ AICc weights < 2 (Table 1), they were not considered competing models and thus model averaging was not applied. The full sex model gave a density estimate of 14.51 leopards/100 km^2^. Female λ_0_ was estimated at 0.05 (± 0.004, 95% CI 0.04–0.06), whilst male λ_0_ was estimated at 0.01 (± 0.002, 95% CI 0.01–0.03). Female σ was estimated at 1584.61 (± 64.56, 95% CI 1460.35–1716.29), and male σ estimated at 2379.76 (± 118.31, 95% CI 2158.94–2623.17).

## 4. Discussion

The reported density of 14.51 leopards/100 km^2^ for the ONR is currently the highest recorded leopard density and one of the first density estimates from SECR analysis in Namibia. Leopard densities vary widely across their range and are dependent on multiple correlates [18]. For leopards occurring outside of fenced, protected reserves in Namibia, relatively low densities have been reported in the literature: 0.9 and 0.59 leopards/100 km^2^ on commercial farmland bordering the eastern boundaries of the Tsau//Khaeb (Sperrgebiet) and Namib-Naukluft National Park respectively, southern Namibia [19]; 1.0 leopards/100 km^2^ in the Waterberg Plateau Park, central Namibia [6]; 1.27 leopards/100 km^2^ in the Bwabwata National Park, north-east Namibia [20] and 3.6 leopards/100 km^2^ on commercial farmland on the south-western border of the Waterberg plateau park [6]. The highest recorded density occurring outside of a protected area in Africa comes from the Soutpansberg Mountains in South Africa at 10.70 leopards/100 km^2^ [21]. Protected, enclosed reserves seem to favor higher population densities than open systems and thus are likely to become increasingly important for the conservation of viable leopard populations [22]. Balme et al. [23] reported a similar high density estimate for the leopard population in the Sabi Sand Game Reserve (11.8 leopards/100 km^2^), an enclosed game reserve in South Africa, as found in the present study area. Studies from other fenced reserves also described higher densities for leopards within closed systems: Brackowski et al. [24] reported a density of 9.28 ± 2.90 leopards/100 km^2^ in Phinda Game Reserve, South Africa and Tarugara at al. [25] reported 61 adult and sub-adult individuals in the 490 km^2^ Malilangwe Wildlife Reserve in Zimbabwe. Within enclosed systems, leopard density has also been found to fluctuate over time, for example during a six-year study within the 85 km^2^ Karongwe Game Reserve, South Africa, Owens [26] recorded leopard density varying between 7.05 and 21.41/100 km^2^. Published literature suggests that enclosed reserves harbour higher densities of leopards than open systems, however many studies have shown that carnivore density is positively correlated to prey abundance [27] and thus results may additionally be a reflexion of the lower prey densities found outside of fenced reserves. Our results add to the growing body of literature which suggests enclosed reserves have the potential to harbour high densities and highlight the importance of such reserves for the survival of threatened species in the future. 

A combination of an unnaturally high prey abundance, the absence of human persecution and a lack of top-down control are believed to be the main drivers of the high leopard population in the ONR. In the presence of larger felids, leopard numbers can be suppressed by top-down process: Lions have been reported to kill leopard cubs, thus repressing the reproductive success of female leopards, as well as adult leopards in the Sabi Sand Game Reserve, South Africa, [28]; tigers (*Panthera tigris*) accounted for a high leopard mortality in the Royal Chitwan National Park in Nepal [29]. Due to the absence of lions in the ONR, leopard numbers are not affected by top-down processes. In contrast, leopards in the ONR have been recorded to be responsible for cheetah mortalities in the study area, and thus may present a top-down controlling factor themselves for subordinate carnivores. During the study period, no leopards were recorded leaving the study area due to the well-fenced boundary, as monitored via VHF radio collars and camera traps. The boundary fence mitigates edge effects and the conflict with humans on surrounding commercial farmland. However, simultaneously, the impermeable fence prevents natural ecological processes, such as immigration and emigration; the absence of these natural processes for maintaining genetic diversity might adversely affect population dynamics [30]. In order to maintain demographic and genetic diversity in an isolated population and to mitigate effects like uncontrolled population growth beyond carrying capacity [31] and inbreeding [32], management guidelines for those populations need to be developed and implemented. These guidelines can include a meta-population management scheme and the introduction of new lineages into the population [33,34] as well as contraception to limit population growth [35], such as that suggested for African wild dogs.

Carrying capacity calculations for leopard are available and can be predicted based on the abundance of their preferred prey [27]. Leopards have a broad diet, with more than one hundred recorded prey species, with prey between 10 and 40 kg being preferred [36]. Based on recorded leopard kills (*n* = 678) warthog (*Phacochoerus africanus*) was the most frequently taken prey (48% of all kills). In contrast, previous studies [36,37,38] have found that prey of the Suidae family, which includes warthog and bushpig (*Potamochoerus larvatus*), are less frequently preyed on by leopards due to their exceeding upper limit of the leopard’s preferred weight range and the ability to inflict serious injuries [36]. Even though warthog numbers are high in the study area, reliable figures are absent, which complicates the robust calculation of preferred prey and carrying capacity of leopards in the ONR. Reliable counts of warthog and other common prey items needed for calculating preferred prey and carrying capacity of leopard within the ONR, should therefore become future management goals. 

## 5. Conclusions

The appliance of camera traps has become an important research tool and is now an integral part of monitoring the leopard population in the ONR, as in other protected areas across Africa. Camera traps allow for a non-invasive method of observation regarding how the population changes over time and how it is impacted by environmental conditions like drought. Here, we have used camera traps to produce the first reliable leopard population estimate for an enclosed reserve in Namibia, whilst adding to the body of literature which suggests some wildlife species in enclosed areas may occur at higher densities than in open systems. Only reliable data received through research, like this study, will have the potential to establish effective conservation and management strategies which are required for the long-term survival of species in an enclosed environment. 

## Figures and Tables

**Table 1:** Model summary table for spatially explicit capture-recaptured models (SECR) models used for estimating leopard density.

Model	Notation	AICc	∆ AICc *	AICwt **	Log-likelihood
Full sex	λ_0_~sex, σ~sex	4220.42	0.00	1.00	−2105.38
σ sex	λ_0_~1, σ~sex	4236.00	15.58	0.00	−2128.02
sex λ0	λ_0_~sex, σ~1	4259.25	38.83	0.00	−2126.15
Null	λ_0_~1, σ~1	4267.33	46.91	0.00	−2291.05
Behaviour	λ_0_~b, σ~1	4269.02	48.60	0.00	−2307.33
Behaviour 2	λ_0_~b, σ~b	4270.34	49.92	0.00	−2309.57

* ∆ AICc is the delta AICc value, the relative difference between the best model (which has a ΔAIC of zero) and each other model in the set. ** AICcwt is the AICc weight, the conditional probabilities for each model.
